# *In Vitro* Antioxidant Capacity of *Opuntia* spp. Fruits Measured by the LOX-FL Method and its High Sensitivity Towards Betalains

**DOI:** 10.1007/s11130-021-00914-7

**Published:** 2021-08-07

**Authors:** Andrea Gómez-Maqueo, Mario Soccio, M. Pilar Cano

**Affiliations:** 1 Biotechnology and Microbiology of Food Department, Institute of Food Science Research (CIAL, CSIC-UAM), Nicolás Cabrera 9, 28049 Madrid, Spain; 2 Escuela de Ingeniería y Ciencias, Tecnologico de Monterrey, Ave. Eugenio Garza Sada 2501, 64700 Monterrey, Mexico; 3 Present Address: Food Structure Team, Clinical Nutrition Research Center, Singapore Institute of Food and Biotechnology Innovation, Agency for Science, Research and Technology, 14 Medical Drive #07-02, MD 6 Building, Yong Loo Lin School of Medicine, Singapore, 117599 Singapore; 4 Department of Agriculture, Food, Natural Resources and Engineering (DAFNE), University of Foggia, Via Napoli, 25, 71122 Foggia, Italy

**Keywords:** Antioxidant capacity, LOX-FL assay, *Opuntia ficus-indica*, *Opuntia stricta* var. *Dillenii*, Betalains, Phenolic compounds

## Abstract

Current *in vitro* methodologies neglect or subestimate the contribution of betalains to antioxidant capacity in foods because they do not reflect their *in vivo* biological mechanisms. In this study, we assessed the sensibility of the lipoxygenase-fluorescein (LOX-FL) method towards betalains, phenolic compounds and ascorbic acid from *Opuntia* spp. fruits; and (ii) the antioxidant capacity of peel and pulp extracts from *Opuntia ficus-indica* L. Mill (var. Fresa, Colorada and Blanco) and *Opuntia stricta var. Dillenii*; by comparing the LOX-FL method to traditional antioxidant methods (ORAC and TEAC). The spectrophotometric monitoring of the LOX-FL reaction avoided interference caused by betalain pigments. Indicaxanthin and betanin showed high antiperoxidative and radical scavenging mechanisms in the LOX-FL assay. *O. stricta *var.* Dillenii* tissues the highest antioxidant capacity which correlated with betanin content. ORAC and TEAC antioxidant methods were less sensible towards betalain antioxidant activity. To our knowledge, this is the first time the LOX-FL antioxidant method has been used on betalains and betalain-rich foods.

**Graphical Abstract:**

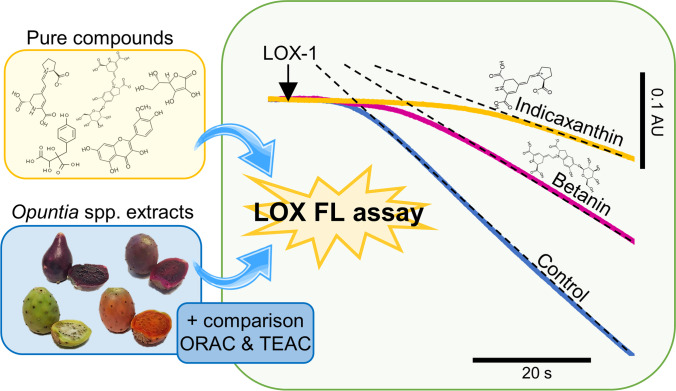

**Supplementary Information:**

The online version contains supplementary material available at 10.1007/s11130-021-00914-7.

## Introduction

Dietary antioxidants are of interest from biological, medical, and nutritional perspectives because they contribute to the reduction of risk of diseases derived from metabolic syndrome. Betalains are water soluble natural colorants composed of a nitrogenous core structure called betalamic acid. They are classified in two groups, betacyanins (red-violet colors) and betaxanthins (yellow-orange colors) depending on the nature of the added residue. The few edible known sources of betalains are red and yellow beetroot (*Beta vulgaris* L. ssp. *vulgaris*), coloured Swiss chard (*Beta vulgaris* L. ssp. *cicla*), grain or leafy amaranth (*Amaranthus* sp.) and cactus fruits, such as those of *Opuntia* and *Hylocereus* genera [1].

Edible cactus from the *Opuntia* genus are nutritious sources of healthy foods adaptable to expanding regions of hot climate [2]. The most widely consumed species from the *Opuntia* genus is prickly pear (*Opuntia ficus-indica* L. Mill.), which is widely cultivated in Latin America, Africa, and in the Mediterranean region (Spain, Italy, Morocco and Algeria). Prickly pear fruits are juicy, colored, fruits rich in betalains (betanin, indicaxanthin, portulacaxanthin, vulgaxanthin, betanidin) and phenolic compounds (piscidic acid, isorhamnetin glycosides, quercetin glycosides and kaempferol glycosides) [3, 4]. The lesser studied variety, *Opuntia stricta *var.* Dillenii*, is native to Central America and may also be found in the Mediterranean region, China, and North Africa. They are referred to as sweet- or wild prickly pear fruits and have recently been characterized in terms of betalains (betanin, phyllocactin, betanidin, and neobetanin) and phenolic compounds (piscidic acid, eucomic acid, and isorhamnetin glycosides) [5, 6].

Betalains have efficient antioxidant action against LOO˙, LO˙, ^1^O2, ˙OH and can prevent lipid peroxidation when incorporated to liposomal bilayers submitted to AAPH [7–10]. They are also good peroxidase substrates for myeloperoxidase and work as one-electron reductants of its redox intermediates [11]. Furthermore, the antioxidant effects of these pigments can prevent or delay the progress of neural death in Parkinson's disease [12] in addition to possessing analgesic activity [13].

However, betalains have low sensitivity to widely used antioxidant assays (*e.g*., ORAC and TEAC) since these *in vitro* assays do not reflect their antioxidant effects *in vivo*. Limitations related to the chemistry of these assays are that they are only able to evaluate scavenging capacity against specific types of radical species (some which are not physiological and biologically relevant) and fail to evaluate other important antioxidant effects. Soybean lipoxygenase-based methods could be suitable alternatives for assessing the antioxidant capacity of betalains because they simultaneously detect the scavenging of physiological radical species, iron ion reducing and chelating activities, and inhibition of the pro-oxidant apoenzyme [14].

The objective of this study was to assess (i) the sensibility of the lipoxygenase-fluorescein (LOX-FL) method towards betalains, phenolic compounds and ascorbic acid from *Opuntia* spp. fruits; and (ii) the antioxidant capacity of peel and pulp extracts from *Opuntia ficus-indica* L. Mill (var. Fresa, Colorada and Blanco) and *Opuntia stricta *var.* Dillenii*; by comparing the LOX-FL method to traditional antioxidant methods (ORAC and TEAC). We expect to shed new light on the antioxidant mechanisms of betanin and indicaxanthin and provide an *in vitro* methodology for assessing the antioxidant capacity of betalain-rich foods.

## Materials and Methods

### Isolated Standards

Betanin-rich extract was obtained from commercial beetroot and purified in a Sephadex L20 resin to obtain the betanin standard [15]. Indicaxanthin was semi-synthesized from purified betalain by raising the pH with ammonia to obtain betalamic acid and by reacting with proline [15]. Piscidic acid was purified by semi-preparative high-performance liquid chromatography (HPLC) from extracts of *Opuntia ficus-indica* peels [16]. Standards for isorhamnetin glucosyl-rhamnosyl-rhamnoside (IG1), isorhamnetin glucosyl-rhamnosyl-pentoside (IG2), isorhamnetin glucosyl-pentoside (IG4) and isorhamnetin glucosyl-rhamnoside (IG5) were isolated from *Opuntia* cladodes [17]. All isolated standards were analyzed by HPLC to determine their purity: betalains (95–100%), piscidic acid (97%) and isorhamnetin glycosides (84–87%). Ascorbic acid was purchased from Sigma-Aldrich (99%).

### Plant Material

Colorada and Fresa prickly pear (*Opuntia ficus-indica* L. Mill.) fruits were obtained from Fasnia (Tenerife, Canary Islands, Spain; 28°2’N, 16°4’W; 446 masl). Blanco prickly pear (*Opuntia ficus-indica* L. Mill.) fruits were obtained from Buenavista del Norte (Tenerife, Canary Islands, Spain; 28°2’N, 16°5’W; 127 masl). Wild prickly pears (*Opuntia stricta *var.* Dillenii*) were obtained from Tinajo (Lanzarote, Canary Islands, Spain; 29°3’N, 13°4’W; 209 masl). Thornless fruits were washed and selected according to uniform maturity, size and no defects. Their physicochemical characteristics were determined in ten fruits of each variety (Supplementary Table S1). Prickly pears were separated into peels and pulps, cut into small pieces (20 × 20 mm), vacuum-sealed in polyethylene bags, frozen with liquid nitrogen and freeze-dried. They were pulverized (Grindomix GM200, Retsch, Germany) to a fine particle size (< 2 mm) and seeds were removed. Pulverized samples were vacuum-sealed and stored at -20 °C until analysis.

### Extracts

Prickly pear extracts were obtained from freeze-dried and pulverized tissues by extracting three times with methanol:water (1:1, v:v), one more time with pure methanol, and by evaporating the methanol from the obtained supernatants to obtain the aqueous extract [16]. The aqueous extracts were analyzed to quantify betalains, phenolic compounds and ascorbic acid and they were used to assess *in vitro* antioxidant capacity by different methods.

### Quantification of Total Betalains by Spectrophotometr*y*

Extracts were diluted and betalains were quantified spectrophotometrically [16] using a SmartSpec Plus BIO-RAD® spectrophotometer and expressed in terms of betaxanthin and betacyanin equivalents by measuring the absorbance at 483 and 535 nm, respectively.

### Quantification of Betalains and Phenolic Compounds by HPLC

Betalains and phenolic compounds were determined simultaneously by high performance liquid chromatography according to reported methodology [3, 16]. The main bioactive compounds were identified by their retention time, UV–visible and mass spectral data compared to those of purified, semi-synthesized and commercial standards (Supplementary Table S3) and quantified using their respective calibration curves. Ascorbic acid was determined by the microplate-adapted colorimetric ascorbate assay [16].

### Lipoxygenase-Fluorescein (LOX-FL) Antioxidant Capacity

Experiments were performed using the LOX/RNO [14] and LOX-FL [18] methods. The LOX/RNO reaction was spectrophotometrically monitored by measuring the absorbance decrease of 4-nitroso-*N*,*N*-dimethylaniline (RNO) at 440 nm in the course of linoleate hydroperoxidation by soybean LOX-1 isoform. The LOX-FL method was performed by fluorometrically monitoring the fluorescein quenching (λ_ex =_ 485 nm; λ_em =_ 515 nm) associated to LOX-1-catalysed linoleate peroxidation.

Based on these results, a protocol for studying the antioxidant capacity of *Opuntia* extracts by the LOX-FL assay was defined. This method involved the spectrophotometric monitoring of the LOX-FL reaction at 485 nm [18] and slight modifications. Fluorescein bleaching was monitored using a spectrophotometer (Specord 210 plus, AnalytikJena, Germany) in a reaction mixture (1 mL) containing 100 mM Na-borate buffer pH 9.0, 400 µM Na-linoleate, 1 µL Tween 20 *per* µmol linoleate and 4.5 µM FL. The linoleate solution was prepared as reported previously. [14] The reaction was started by adding 0.5 EU of soybean lipoxygenase. Measurements were carried out in both the absence (control) and presence of sample (extract or standard). The rate of the reaction expressed as ΔA_485_ ∙ min^−1^, was calculated as the highest slope to the experimental curve. The lag phase was calculated as the time occurring between enzyme addition to the test sample and the start of the reaction.

The inhibition of the LOX-FL reaction was determined by calculating the decrease of the rate of the fluorescein bleaching in the presence of sample (extract or standard) (v_a_) with respect to the control (v_c_), according to the equation: Inhibition (%) = [1-(v_a_/v_c_)]∙100. Antioxidant capacity was calculated by means of a dose–response curve obtained with Trolox by plotting the decrease of the rate of fluorescein bleaching as a function of the Trolox concentration according to the equation: Inhibition (%) = 4.8594 [Trolox] + 11.6 (r = 0.9942, 

*p* < 0.001), where the concentration of Trolox was 2–10 µM. Since both the rate and the lag phase of the LOX-FL reaction were affected by methanol, when isolated isorhamnetin glycosides and piscidic acid (reconstituted in methanol) were evaluated, a constant volume of 50 µL of methanol was also maintained in the control.

### TEAC Antioxidant Capacity

The TEAC assay was analyzed spectrophotometrically at 734 nm [19]. The radical cation ABTS + (2,2'-azino-bis-3-ethylbenzothiazoline-6-sulfonic acid) was generated by ABTS oxidation with potassium persulfate. The ABTS• + solution was diluted with 5 mM Na-phosphate buffer at pH 7.4 to obtain an initial absorbance value at 734 nm (A_734_) of 0.70 ± 0.20. The assay contained 1.0 mL of the ABTS• + diluted solution, the sample (extract or standard), and Na-phosphate buffer (pH 7.4) to obtain a final volume of 1.1 mL assay. Absorbance at 734 nm was read 5 min after. The decrease of absorbance (%) with respect to the blank was used to quantify antioxidant capacity using a concentration–response Trolox curve 10–150 µM.

### ORAC Antioxidant Capacity

The ORAC protocol [20] was performed by measuring fluorescence degradation in a 96-well microplate. Every working well of a 96-well plate containing the assay mixture (final volume 0.2 mL) consisting of 75 mM Na-phosphate buffer (pH 7.4), 7 µM fluorescein and an appropriate volume of sample (extract or standard). The reaction was started by adding 46 mM AAPH (2,2'-azobis (2-amidinopropane) dihydrochloride) in the well. Fluorescence intensity decay was monitored once every minute during 60 min at 37 °C. Monitoring was done at 485 nm excitation and 530 nm emission wavelengths. To quantify antioxidant capacity, the difference between the area under the fluorescence decay kinetic curve (area under curve, AUC) of sample and the AUC of the blank was calculated. Antioxidant capacity was determined using a Trolox dose–response curve (10–80 µM) repeated each time the assay was performed.

### Statistical Analysis

Results were expressed as mean ± standard deviation (*n* = 3). Significant differences were calculated by one-way analysis of variance (ANOVA), followed by *post hoc* Duncan’s test (*p* < 0.05). Pearson’s correlation coefficients (*r*) were determined (*p ≤ 0.05, **p ≤ 0.001, bilateral, *n* = 24). Statistical analysis was determined with SPSS Statistics 23.0 (IBM Corp, Armonk, USA).

## Results and Discussion

### Characterization of *Opuntia* spp. Fruit Extracts by HPLC

Extracts from *Opuntia ficus-indica* (var. Colorada, Fresa and Blanco) and *Opuntia stricta *var.* Dillenii* peels and pulps were characterized in terms of betalains, phenolic compounds and ascorbic acid (Supplementary Table S2). The extensive betalain and phenolic profile in *O. ficus-indica* fruits (var. Fresa, Colorada and Blanco) from the Canary Islands has been previously determined [3] and the *O. stricta *var.* Dillenii* betalain and phenolic profile was tentatively evaluated in our laboratory in this work.

### Suitability of Soybean Lipoxygenase-Based Antioxidant Methods

**Fig. 1:**
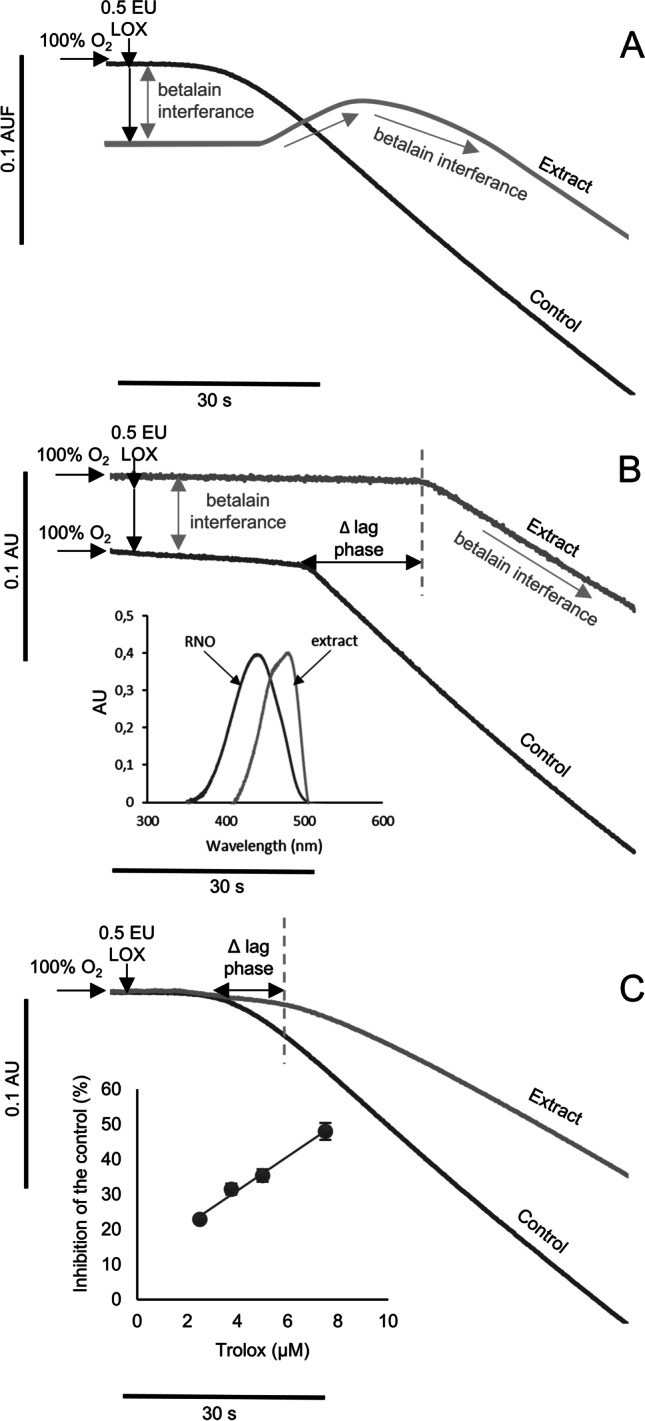
Comparison of soybean-lipoxygenase methods in betalain-rich extracts and standards. (**A**) LOX-FL method (fluorometric monitoring), (**B**) LOX/RNO method (spectrophotometric monitoring) and RNO and extract UV–Vis spectra, (**C**) LOX-FL method (spectrophotometric monitoring) and Trolox calibration curve. Δ lag phase: lag phase change in the presence of extract

The suitability of the LOX-FL reaction was assessed by evaluating its sensitivity to *Opuntia* spp. fruit extracts and isolated betalains. We studied the interference of these pigments (due to their spectral properties) by LOX-FL and LOX/RNO methodologies (Fig. 1). Following the traditional LOX-FL method (fluorometric monitoring), a high concentration of betalains (λ_abs_ 480 and 530 nm) may quench fluorometric values (λ_exc_ 485 nm and λ_emi_ 515 nm) during the assay (Fig. 1A). Meanwhile, using the LOX/RNO assay (Fig. 1B) betalains caused false positive values by increased the absorbance of the reaction.

The LOX-FL reaction monitored spectrophotometrically at 485 nm was the best method to assess the antioxidant capacity of betalains and *Opuntia* spp. extracts (Fig. 1C). This method has been previously used to evaluate the kinetic properties of the soybean-lipoxygenase reaction [18], being able to replace the detection by fluorimetry. Interestingly, we found that this approach required lower amounts of betalains compared to the LOX/RNO method, by reducing the interference on the assay which was negligible.

### Antioxidant Capacity by the LOX-FL Assay

**Fig. 2:**
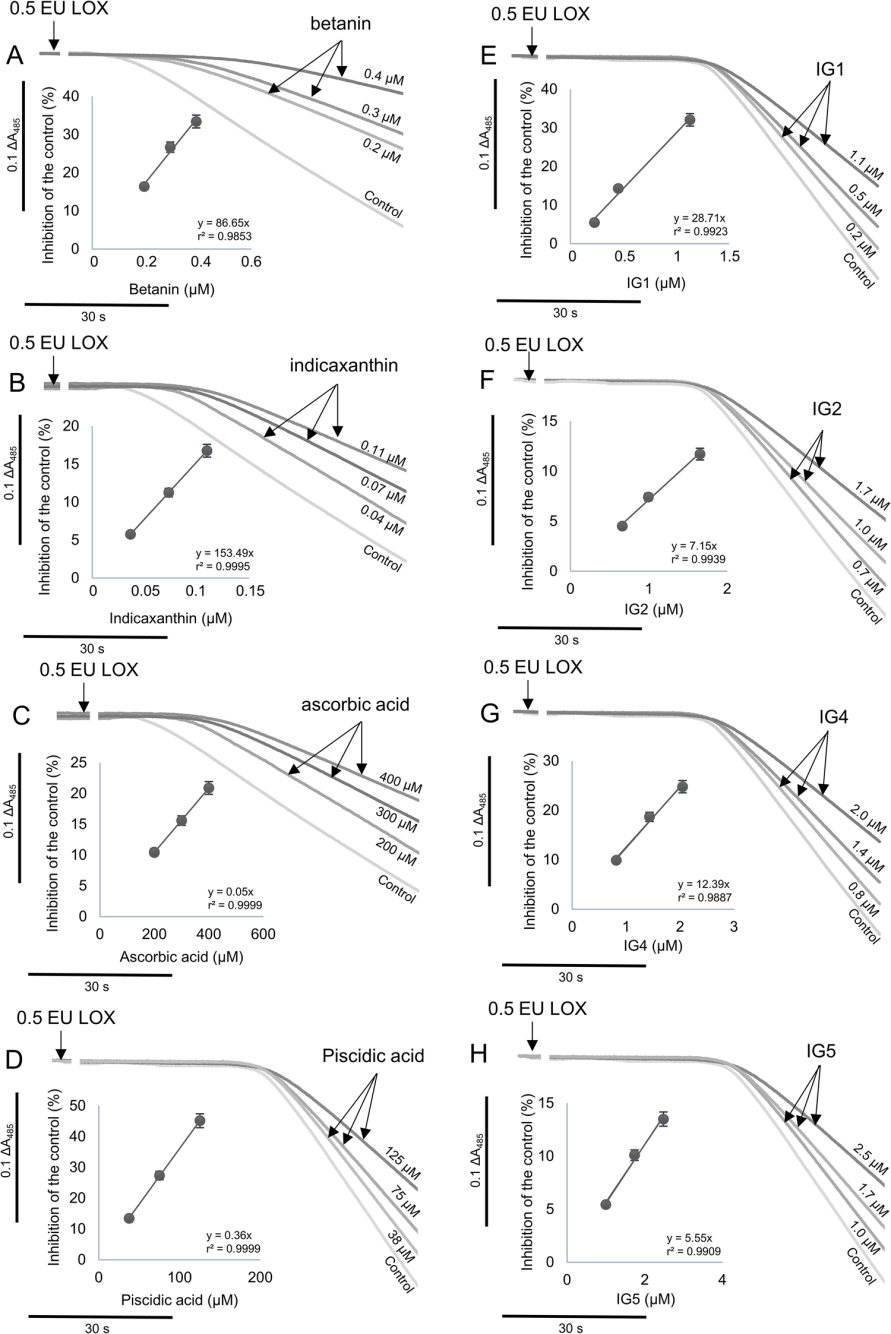
Inhibition of the LOX-1-dependent fluorescein bleaching by (**A**) betanin, (**B**) indicaxanthin, (**C**) ascorbic acid, (**D**) piscidic acid, (**E**) isorhamnetin glucosyl-rhamnosyl-rhamnoside (IG1), (**F**) isorhamnetin glucosyl-rhamnosyl-pentoside (IG2), (**G**) isorhamnetin glucosyl-pentoside (IG4) and (**H**) isorhamnetin glucosyl-rhamnoside (IG5)

Isolated betalain, phenolic compound and ascorbic acid standards were individually assessed (Fig. 2) by the LOX-FL antioxidant method. The LOX-FL reaction consists of two stages [14]: i) a lag phase, representing the time necessary to consume the oxygen in the reaction mixture due to the primary LOX-1 reaction of linoleate hydroperoxidation (aerobic cycle), and ii) a bleaching phase of fluorescein, due to the LOX-1 mediated generation of physiologically relevant radical species (mainly alkoxyl, peroxyl and hydroxyl radicals), occurring when anaerobiosis is reached in the assay mixture (anaerobic cycle). All standards showed a positive linear dependence between reaction inhibition and compound concentration. However, the different behavior with respect to the lag phase and bleaching rate of fluorescein strongly suggested different antioxidant actions.

On one hand, betalains (betanin, indicaxanthin) and ascorbic acid inhibited the rate of the fluorescein bleaching (anaerobic reaction), showing radical scavenging activity. Additionally, they increased the lag phase of the reaction (aerobic reaction) evidencing their antiperoxidative action (Fig. 2A-C; Table 1). On the other hand, phenolic compounds (piscidic acid and isorhamnetin glycosides) inhibited the reaction rate by showing high radical scavenging activity but did not affect the lag phase of the reaction (Fig. 2D-H).

**Table 1:** Antioxidant capacity of individual standards (betalains, phenolic compounds and ascorbic acid) by means of the LOX-FL method

	LOX-FL		ORAC^2^	TEAC
Pure compound	µmol Trolox eq/mg	Δ lag phase^1^	µmol Trolox eq/mg	µmol Trolox eq/mg
Betalains				
Indicaxanthin	104.00 ± 1.98^a^	+ + +	17.32 ± 0.68^c^	9.04 ± 2.10^a^
Betanin	32.27 ± 1.40^b^	+ +	10.92 ± 0.44^b^	5.17 ± 0.52^a^
Phenolic acid				
Piscidic acid	0.290 ± 0.002^ef^	-	3.56 ± 0.16^a^	6.43 ± 0.74^a^
Flavonoid glycosides				
IG1 (Isorhamnetin glucosyl-rhamnosyl-rhamnoside)	7.64 ± 0.99^c^	-	34.96 ± 1.4^e^	122.7 ± 8.59^c^
IG2 (Isorhamnetin glucosyl-rhamnosyl-pentoside)	1.90 ± 0.09^e^	-	30.24 ± 1.2^d^	139.56 ± 6.98^c^
IG4 (Isorhamnetin glucosyl-pentoside)	3.94 ± 0.23^d^	-	27.76 ± 1.12^d^	124.78 ± 13.20^c^
IG5 (Isorhamnetin glucosyl-rhamnoside)	1.79 ± 0.12^e^	-	18.00 ± 0.72^c^	72.95 ± 3.54^b^
Organic acid				
Ascorbic acid	0.0610 ± 0.0003^f^	+	3.04 ± 0.12^a^	8.95 ± 1.00^a^

Means ± standard deviation (*n *= 3). Different letters indicate statistically significant differences (*p* ≤ 0.05) according to Duncan’s test. ^1^Relative lag phase change (s/mg standard). ^2^Reported in Gómez-Maqueo et al. (2019). LOX-FL values correspond to the experimental data shown in Fig. 2

In Table 1, the antioxidant capacity of pure indicaxanthin, betanin, isorhamnetin glycosides (IG1, IG2, IG4 and IG5), piscidic and ascorbic acids determined by the LOX-FL antioxidant assay (spectrophotometric monitoring) and by ORAC and TEAC antioxidant methods are shown.

In the LOX-FL method, indicaxanthin had the highest antioxidant capacity (104 μmol Trolox eq./mg) followed by betanin (32 μmol Trolox eq./mg). Though considerably lower than betalains, isorhamnetin glycosides also showed high LOX-FL antioxidant capacity. Piscidic acid was the less active phenolic compound. A low antioxidant capacity value was observed for ascorbic acid. Furthermore, the lag phase of the reaction was affected by betanin, indicaxanthin and ascorbic acid. These results agree with previous findings that show the antioxidant performance of betalains can be higher than that of several flavonoids, ascorbic acid, and tocopherols [21].

Regarding the ORAC antioxidant capacity assay, isorhamnetin glycoside IG1 showed the highest antioxidant capacity (35 μmol Trolox eq./mg) followed by IG2 and IG4 (28 – 30 μmol Trolox eq./mg) and IG5 (18 μmol Trolox eq./mg). This data has been previously reported [14] where the antioxidant capacity of isorhamnetin glycosides was found to be 3.2–6.1 times the activity of the isorhamnetin aglycone. The ORAC antioxidant method is an indicator of the peroxyl radical scavenging capacity of antioxidants and measures their hydrogen atom donating ability (HAT-based method).

In the TEAC antioxidant method, isorhamnetin glycosides IG1, IG2 and IG4 all showed high antioxidant capacity values (123–140 μmol Trolox eq./mg). Meanwhile, indicaxanthin, betanin, ascorbic acid and piscidic acid all showed the lowest antioxidant capacity (6–9 μmol Trolox eq./mg). The TEAC antioxidant method measures the ability of antioxidants to scavenge the stable radical cation ABTS ^+^ by single electron transfer (SET-based method).

### Putative Antioxidant Mechanisms of Betalains in the LOX-FL Assay

Soybean lipoxygenase (LOX)-1 assays are based on the aerobic and anaerobic reaction of the enzyme in the presence of linoleic acid. A key role in LOX-1 catalysis is played by non-heme iron atom, cycling from the oxidized form (III) to the reduced one (II). When the main aerobic cycle consumes oxygen in the reaction mixture, the anaerobic cycle starts and different physiological reactive species, including the peroxyl (LOO∙), alkoxyl (LO∙), hydroxyl (∙OH) and alkylic (L∙) radicals as well as the singlet oxygen (^1^O_2_) are generated. These oxidant species induce the bleaching of fluorescein. Interestingly, the soybean LOX-1-catalysed FL-bleaching may be delayed, inhibited or even prevented by antioxidants acting according to different mechanisms, such as the capacity to scavenge one or more free radical species, as well as other antioxidant mechanisms involving chelating or reducing activities of iron ion essential for LOX-1 catalysis, singlet oxygen quenching, hydroperoxide decomposition, and direct inhibition of pro-oxidative LOX-1 apo-enzyme.

Betanin and betanidin (betacyanins) inhibit the primary aerobic reaction of LOX-1 isoenzyme at very low concentrations, with IC_50_ values of 0.25 and 0.5 μM, respectively [7]. This suggests that the strong inhibition observed by betalains in the LOX-FL assay was achieved through reduction of iron to the ferrous inactive form and/or by interacting with the enzyme peroxyl radical complex. This data agrees with our results reported in Fig. 2A in which a marked increase of lag phase of the LOX-FL reaction due to betanin is shown. A similar mechanism could be supposed for indicaxanthin (betaxanthin) due to its similar chemical structure which has also shown ferric reducing antioxidant power [22]. In the light of their putative reducing power, betanin and indicaxanthin could inhibit LOX-1 also at level of the anaerobic cycle, where a key role of non-heme iron atom in LOX-catalysis is also played.

Moreover, efficient antioxidant action against LOO·, LO·, ^1^O_2_, ·OH and the ability to prevent lipid peroxidation when incorporated into liposomal bilayers submitted to AAPH, have been also reported for betanin [7–9] and indicaxanthin [10]. This data agrees with the findings in Fig. 2A-B where, in the presence of betanin and indicaxanthin, a significant reduction in the rate of fluorescein bleaching can be observed. Interestingly, the presence of glycosylation and further acylation in the chemical structures of betalains have been reported to reduce the radical scavenging activity mainly of betacyanins (such as betanin) [23]. This evidence is further supported by our results by the LOX-FL method (Table 1), which highlight a higher antioxidant capacity for indicaxanthin than for betanin.

### Comparison of the Antioxidant Capacity in Extracts by LOX-FL, ORAC and TEAC Methods

**Fig. 3:**
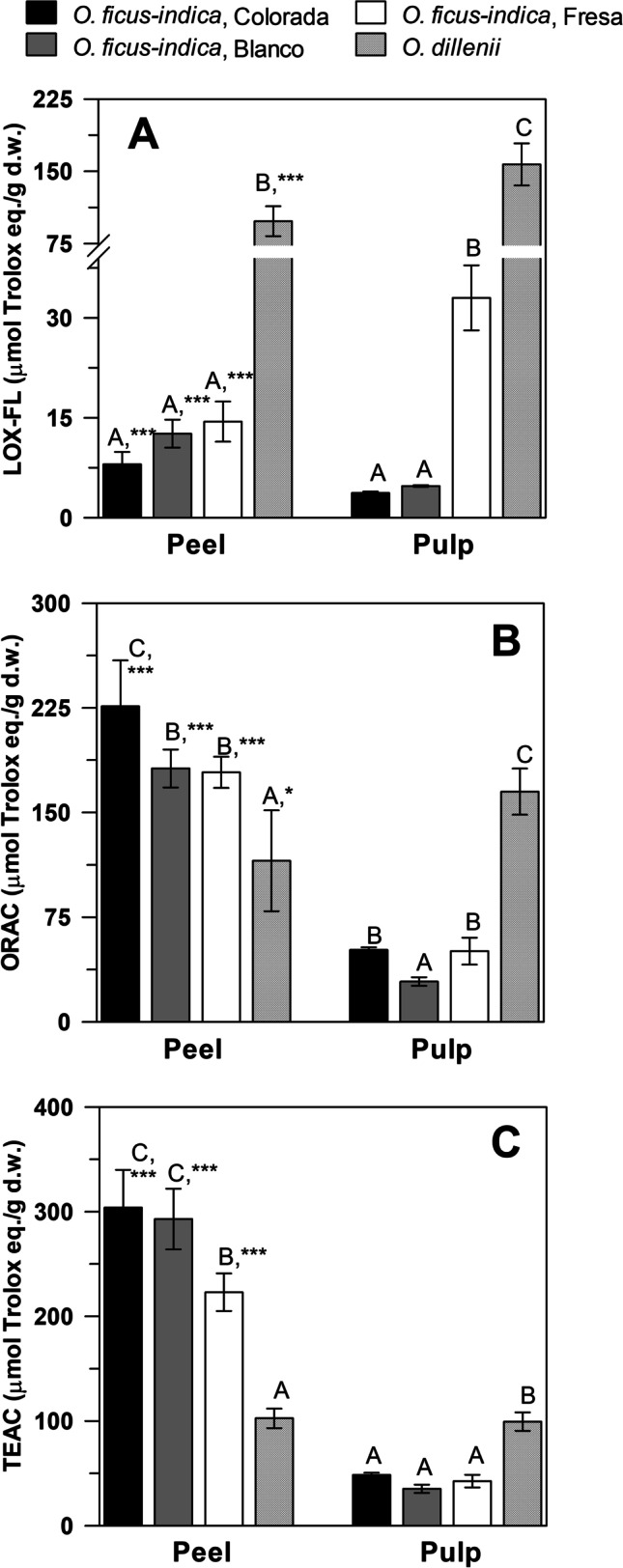
Antioxidant capacity of *Opuntia* spp. fruit extracts by (**A**) LOX-FL, (**B**) ORAC and (**C**) TEAC methods. Letters indicate statistically significant differences (*p* < 0.01) between genotypes. *, *** indicate the probability level at* P* ≤ 0.05 and *P* ≤ 0.001, respectively, relative to the comparison between peel and pulp values. Raw data may be consulted in Supplementary Table S4

The antioxidant capacity of peel and pulp extracts from *O. ficus-indica* and *O. stricta *var.* Dillenii* fruits were evaluated by the LOX-FL spectrophotometric protocol and compared to the widely used ORAC and TEAC assays (Fig. 3; Supplementary Table S4). TEAC and ORAC antioxidant assays mainly assess the reducing power capacity and scavenging activity against peroxyl radicals, respectively; whereas the LOX-FL method simultaneously detects scavenging capacity against different physiological radicals as well as other antioxidant functions, thus providing a more comprehensive result on antioxidant capacity [18, 23].

The LOX-FL, ORAC and TEAC antioxidant assays provided different results for aqueous extracts of *Opuntia* spp. fruits in the light of their different mechanisms of detection. Interestingly, all three assays agreed in highlighting a significant higher antioxidant efficacy for pulp extracts from *O. stricta var. Dillenii* fruit tissues. However, tendencies in peels were significantly different in the LOX-FL assay compared to ORAC and TEAC antioxidant methods. The antioxidant capacity in prickly pear peels by ORAC and TEAC was highest in the fruits with the highest phenolic content (Colorada and Blanca *O. ficus-indica* fruits), particularly isorhamnetin glycosides. In the LOX-FL method of peels, *O. stricta *var.* Dillenii* had the highest antioxidant capacity due to its high betanin content. Meanwhile, *O. ficus-indica* varieties had higher antioxidant capacity measured by TEAC and ORAC antioxidant methods. It should be emphasized that the *O. ficus-indica* fruit varieties generally showed a higher antioxidant performance in peels than pulps (with the only exception of Fresa measured by LOX-FL). While in *O. dilleni,* the antioxidant effectiveness of the peel was lower (measured by LOX-FL and ORAC), or at most similar, (measured by TEAC) than its pulp.

### Correlation Analysis Between Antioxidant Capacity and Antioxidant Content in Extracts

Pearson’s correlation between the antioxidant capacity of all *Opuntia* spp. peel and pulp tissues by LOX-FL, ORAC and TEAC assays and their bioactive content (individual betalains, phenolic compounds and ascorbic acid) are shown in Supplementary Table S5.

The antioxidant capacity of *Opuntia* spp. extracts determined by the LOX-FL method had strong correlation with betanin (r = 0.90), isobetanin (r = 0.93), and total betalain (r = 0.89) content at *p* ≤ 0.01. However, no significant correlation was found between the LOX-FL method and indicaxanthin content, possibly due to the scarce indicaxanthin-rich *O. ficus-indica* varieties included in the study (only Colorada). We suggest further studies comparing the antioxidant capacity of several indicaxanthin-rich plant foods by the LOX-FL reaction.

Both ORAC and TEAC assays correlated (*p* ≤ 0.01) with phenolic compounds, namely, piscidic acid, IG1, IG2 and IG4. ORAC and TEAC methods can detect hydrogen atom transfer and single electron transfer, respectively, which are well-established antioxidant mechanisms for phenolic compounds. However, ORAC and TEAC antioxidant methods did not correlate with betalains in *Opuntia* spp. fruits, despite their abundance. In the literature, there are inconsistent results regarding the correlation of these assays with betalain content.

None of the antioxidant assays used in this study correlated with ascorbic acid content. Nevertheless, in the light of its high reducing and radical scavenging capacities, ascorbic acid could contribute to prickly pears´ antioxidant capacity by protecting other antioxidant constituents from oxidative damage.

As for comparison among the different antioxidant capacity assays, a positive correlation (0.895, *p* ≤ 0.01) was found between TEAC and ORAC results, whereas the LOX-FL method appeared unrelated to ORAC and TEAC.

## Conclusions

The correlation between antioxidant activity by the LOX-FL assay and betalain content suggest the effectivity of this *in vitro* method to reflect the *in vivo* antioxidant capacity of betalains. This is attributable to the capability of these compounds to affect the aerobic and anaerobic reactions involved in the LOX-FL assay: i) inhibition of LOX-1 through conversion to its ferrous inactive form and interaction with the enzyme-peroxyl radical-complex, and ii) scavenging of peroxyl, alkoxyl, hydroxyl radicals. We expect this method could be used in the future to analyze the antioxidant activity of betalain-rich foods because of its biological relevance.

## Supplementary Information

Below is the link to the electronic supplementary material.

Supplementary file1 (PDF 130 KB)

Supplementary file2 (PDF 96 KB)

Supplementary file3 (PDF 88 KB)

Supplementary file4 (PDF 70 KB)

Supplementary file5 (PDF 83 KB)

## Data Availability

All data generated or analyzed during this study are included in this published article and its supplementary information files.

## Abbreviations

LOX-FL: Lipoxygenase-fluorescence
ORAC: Oxygen radical absorbance capacity
TEAC: Trolox equivalent antioxidant capacity
