# Supplementary material for: In Vitro Antioxidant Capacity of Opuntia spp. Fruits Measured by the LOX-FL Method and its High Sensitivity Towards Betalains
Source: Plant Foods Hum Nutr. 2021 Aug 7;76(3):354–62. doi: 10.1007/s11130-021-00914-7 (PMC8426225; doi:10.1007/s11130-021-00914-7)
Supplement: Supplementary file 1 — Supplementary file1 (PDF 130 KB) [file 11130_2021_914_MOESM1_ESM.pdf]

Supplementary Table S1. Physicochemical characteristics and total betalains (mg betalain eq./g d.w.; spectrophotometric determination) of *O. stricta var. Dillenii* and *O. ficus-indica* (var. Fresa, Colorada and Blanco) fruits.

	**Tissue**	***O. stricta var. Dilleni***	***O. ficus-indica***		
			**Fresa**	**Colorada**	**Blanco**
**Physicochemical characteristics**					
Physical appearance	Whole fruit	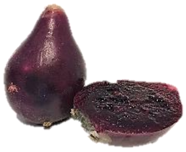	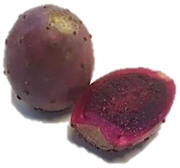	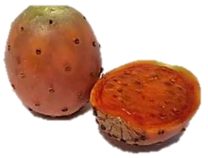	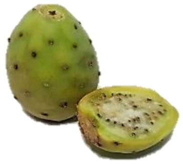
Color	peel	purple	fuchsia	orange	green
	pulp	purple	fuchsia	orange	white
Apical caliber (cm)	whole fruit	6.1 ± 0.6^{a}	5.8 ± 0.5^{a}	6.2 ± 0.8^{a}	6.6 ± 0.7^{a}
Equatorial caliber (cm)	whole fruit	4.0 ± 0.2^{a}	4.5 ± 0.4^{a}	4.9 ± 0.3^{b}	5.1 ± 0.3^{b}
Weight (g)	whole fruit	54.4 ± 6.8^{a}	78.9 ± 9.6^{ab}	106.0 ± 9.3^{bc}	129.6 ± 23.9^{c}
Wet basis moisture (%)	peel	83.4 ± 2.5^{a}	89.1 ± 0.9^{a}	88.2 ± 2.6^{a}	90.6 ± 3.9^{a}
	pulp	79.1 ± 3.0^{a}	85.6 ± 2.2^{a}	86.1 ± 2.4^{a}	83.6 ± 1.9^{a}
Soluble solids (°Brix)	pulp	10.8 ± 0.3^{a}	13.6 ± 0.1^{b}	13.4 ± 0.1^{b}	15.7 ± 0.1^{c}
pH	pulp	3.6 ± 0.1^{a}	6.1 ± 0.0^{b}	6.1 ± 0.2^{b}	6.6 ± 0.1^{c}
**Total betalains (spectrophotometric determination)**					
Total betaxanthins[1]	peel	2.40 ± 0.02^{a}	0.34 ± 0.05^{b}	0.27 ± 0.02^{b}	0.17 ± 0.06^{c}
	pulp	$3.68 \pm 0.35^{a**}$	0.50 ± 0.19^{b}	$0.20 \pm 0.02^{c**}$	0.15 ± 0.01^{c}
Total betacyanins[2]	peel	6.35 ± 0.5^{a}	0.72 ± 0.08^{c}	1.03 ± 0.03^{b}	0.18 ± 0.09^{d}
	pulp	6.71 ± 0.46^{a}	0.67 ± 0.35^{b}	$1.18 \pm 0.05^{b*}$	0.75 ± 0.5^{b}
Total betalains[3]	peel	8.75 ± 0.25^{a}	1.06 ± 0.13^{b}	1.31 ± 0.05^{b}	0.35 ± 0.15^{c}
	pulp	$10.35 \pm 0.81^{a*}$	1.18 ± 0.54^{b}	1.37 ± 0.06^{b}	0.91 ± 0.49^{b}

Physicochemical characteristics represent mean ± standard deviation (n=10). Total betalains determined spectrophotometrically represent mean ± standard deviation (n=3). Superscript letters indicate statistically significant differences ($p \leq 0.05$) by Duncan's test. * and ** indicate the probability level at $p \leq 0.05$ and $p \leq 0.01$, respectively, relative to the comparison between peel and pulp values according to the Student's t-test. [1]mg indicaxanthin eq./g d.w.; [2]mg betanin eq./g d.w.; [3]algebraic sum of betaxanthins and betacyanins.
