# Supplementary material for: In Vitro Antioxidant Capacity of Opuntia spp. Fruits Measured by the LOX-FL Method and its High Sensitivity Towards Betalains
Source: Plant Foods Hum Nutr. 2021 Aug 7;76(3):354–62. doi: 10.1007/s11130-021-00914-7 (PMC8426225; doi:10.1007/s11130-021-00914-7)
Supplement: Supplementary file 2 — Supplementary file2 (PDF 96 KB) [file 11130_2021_914_MOESM2_ESM.pdf]

Supplementary Table S2. Quantification of individual betalains and phenolic compounds (µg/g d.w.) by HPLC and ascorbic acid content (µg/g d.w.) in peels and pulps of *O. stricta var. Dillenii* and *O. ficus-indica* (var. Fresa, Colorada and Blanco) fruits.

**Compound**	**Tissue**	***O. stricta var. Dillenii***	***O. ficus-indica***		
			**Fresa**	**Colorado**	**Blanco**
Betanin	peel	6008.1 ± 55.7^{cB}	954.1 ± 59.4^{bA}	54.1 ± 2.0^{aA}	2.2 ± 0.2^{aA}
	pulp	4771.5 ± 111.1^{cA}	1117.3 ± 27.8^{bA}	61.5 ± 2.0^{aA}	2.6 ± 0.4^{aA}
Isobetanin	peel	527.8 ± 22.5^{cA}	50.9 ± 9.4^{bA}	7.1 ± 0.4^{aA}	n.d.^{aA}
	pulp	466.9 ± 8.0^{cA}	102.2 ± 11.9^{bB}	5.3 ± 1.1^{aA}	tr.^{aA}
Indicaxanthin (Bx-proline)	peel	n.d.^{aA}	123.5 ± 6.4^{bA}	598.1 ± 19.2^{cA}	2.8 ± 0.1^{aA}
	pulp	n.d.^{aA}	400.6 ± 14.0^{bB}	1179.4 ± 14.8^{cB}	5.0 ± 0.2^{aB}
Piscidic acid	peel	6976.6 ± 6.1^{aB}	39688.2 ± 998.9^{bB}	40735.2 ± 1344.2^{bB}	42360.6 ± 3796.2^{bB}
	pulp	2981.6 ± 79.8^{aA}	3653.2 ± 19.9^{bA}	4749.0 ± 4.6^{cA}	4633.0 ± 58.3^{cA}
IG1 (isorhamnetin glucosyl-rhamnosyl-rhamnoside)	peel	41.5 ± 6.1^{aA}	217.3 ± 18.9^{dB}	179.6 ± 2.9^{cB}	111.3 ± 1.7^{bB}
	pulp	24.0 ± 1.8^{bA}	2.2 ± 0.2^{aA}	1.1 ± 0.0^{aA}	2.7 ± 0.2^{aA}
IG2 (isorhamnetin glucosyl-rhamnosyl-pentoside)	peel	10.1 ± 3.0^{aA}	220.8 ± 21.0^{dB}	160.5 ± 2.7^{cB}	53.1 ± 0.8^{bB}
	pulp	3.3 ± 0.1^{dA}	1.8 ± 0.2^{cA}	0.6 ± 0.1^{aA}	1.2 ± 0.1^{bA}
IG4 (isorhamnetin glucosyl-pentoside)	peel	36.8 ± 2.3^{bB}	138.4 ± 5.6^{dB}	94.5 ± 0.5^{cB}	16.2 ± 4.5^{aB}
	pulp	2.1 ± 0.2^{bA}	0.6 ± 0.2^{aA}	0.2 ± 0.1^{aA}	0.5 ± 0.1^{aA}
IG5 (isorhamnetin glucosyl-rhamnoside)	peel	600.8 ± 7.8^{dB}	321.1 ± 6.8^{cB}	284.5 ± 7.2^{bB}	115.0 ± 5.3^{aB}
	pulp	23.8 ± 0.1^{dA}	1.5 ± 0.2^{bA}	0.8 ± 0.1^{aA}	4.7 ± 0.4^{cA}
Ascorbic acid	peel	10493.7 ± 298.9^{bA}	2435.2 ± 28.1^{aB}	2536.0 ± 160.9^{aB}	2357.7 ± 70.1^{aB}
	pulp	9831.3 ± 326.9^{bA}	1404.0 ± 38.0^{aA}	1347.0 ± 52.6^{aA}	1570.7 ± 65.5^{aA}

Data represent mean ± standard deviation (n=3). Different lowercase superscript letters indicate statistically significant differences ($p \leq 0.05$) between varieties by Duncan's test. Different uppercase superscript letters indicate statistically significant differences ($p \leq 0.05$) between the peel and pulp of each variety by student's t-test. n.d. not detected, tr. traces.
