# Supplementary material for: In Vitro Antioxidant Capacity of Opuntia spp. Fruits Measured by the LOX-FL Method and its High Sensitivity Towards Betalains
Source: Plant Foods Hum Nutr. 2021 Aug 7;76(3):354–62. doi: 10.1007/s11130-021-00914-7 (PMC8426225; doi:10.1007/s11130-021-00914-7)
Supplement: Supplementary file 3 — Supplementary file3 (PDF 88 KB) [file 11130_2021_914_MOESM3_ESM.pdf]

Supplementary Table S3. HPLC retention times, UV/Vis spectra and MS spectral data of betalains and phenolic compounds in prickly pear (*O. ficus-indica*) and wild prickly pear (*O. stricta var. Dillenii*) fruits.

Rt (min)	Compound Identity	UV λ_{max} (nm)	$[M+H]^+$ (m/z)	MS/MS (m/z)
**Betalains**				
10.5	Indicaxanthin (Bx-proline)[a]	478	309.11	263.10, 217.10, 70.06
15.7	Betanin[a]	534	551.15	390.10, 389.10
20.7	Isobetanin[b]	534	551.15	390.10, 389.10
**Phenolic acid**				
14.0	Piscidic acid[a]	232, 275	257.07	191.07, 147.04, 119.05, 107.05
**Flavonoid glycosides**				
40.0	IG1 (isorhamnetin glucosyl-rhamnosyl-rhamnoside)[a]	254, 354	771.23	625.18, 317.07, 85.03
40.4	IG2 (isorhamnetin glucosyl-rhamnosyl-pentoside)[a]	253, 354	757.22	317.07, 167.07, 86.10
41.2	IG4 (isorhamnetin glucosyl-pentoside)[a]	254, 354	611.16	479.12, 317.07, 177.05
44.5	IG5 (isorhamnetin glucosyl-rhamnoside)[a]	253, 354	625.18	317.07, 85.03

[a]Confirmed and quantified with semi-synthesized, purified, or commercial standard. [b]Quantified using betanin calibration curve.
