# Supplementary material for: In Vitro Antioxidant Capacity of Opuntia spp. Fruits Measured by the LOX-FL Method and its High Sensitivity Towards Betalains
Source: Plant Foods Hum Nutr. 2021 Aug 7;76(3):354–62. doi: 10.1007/s11130-021-00914-7 (PMC8426225; doi:10.1007/s11130-021-00914-7)
Supplement: Supplementary file 4 — Supplementary file4 (PDF 70 KB) [file 11130_2021_914_MOESM4_ESM.pdf]

Supplementary Table S4. Antioxidant capacity (µmol Trolox eq./ g dry weight) of peels and pulps of prickly pear (*O. ficus-indica*) and wild prickly pear (*O. stricta var. Dillenii*) fruit varieties by (A) LOX-FL, (B) ORAC and (C) TEAC methods.

**Antioxidant capacity**[1]	**Tissue**	***O. stricta var. Dillenii***	***O. ficus-indica***		
			**Fresa**	**Colorada**	**Blanco**
LOX-FL	peel	98.2±15.6^{a**}	14.4±3.0^{b**}	8.04±1.84^{b**}	12.6±2.1^{b**}
	pulp	157.1±21.8^{a}	33.0±4.9^{b}	3.71±0.20^{c}	4.75±0.13^{c}
ORAC	peel	115.4±36.1^{c*}	178.8±1.2^{b**}	226.1±32.9^{a**}	181.6±13.6^{b**}
	pulp	165.0±16.5^{a}	50.7±9.7^{b}	51.6±1.9^{b}	28.9±3.1^{c}
TEAC	peel	102.6±9.3^{c}	223±18^{b**}	304±36^{a**}	293±29^{a**}
	pulp	99.5±8.9^{a}	42.7±6.0^{b}	48.4±2.1^{b}	35.3±4.0^{b}

Values are the means of three independent determinations ± standard deviation. Different letters indicate statistically significant differences ($p < 0.01$) between genotypes according to Duncan's test. * and ** indicate the probability level at $p \leq 0.05$ and $p \leq 0.001$, respectively, relative to the comparison between peel and pulp values according to the Student's *t*-test.[1]µmol Trolox eq./ g dry weight
