# Supplementary material for: In Vitro Antioxidant Capacity of Opuntia spp. Fruits Measured by the LOX-FL Method and its High Sensitivity Towards Betalains
Source: Plant Foods Hum Nutr. 2021 Aug 7;76(3):354–62. doi: 10.1007/s11130-021-00914-7 (PMC8426225; doi:10.1007/s11130-021-00914-7)
Supplement: Supplementary file 5 — Supplementary file5 (PDF 83 KB) [file 11130_2021_914_MOESM5_ESM.pdf]

Supplementary Table S5. Pearson's correlation coefficients (*r*) between antioxidant activity (LOX-FL, ORAC and TEAC) and bioactive compounds in *O. ficus-indica* and *O. stricta var. Dillenii* peel and pulp extracts.

	(1)	(2)	(3)	(4)	(5)	(6)	(7)	(8)	(9)	(10)	(11)	(12)	(13)	(14)
(1) LOX-FL	1													
(2) ORAC	.167	1												
(3) TEAC	-.233	.895**	1											
(4) Indicaxanthin	-.425	-.211	-.133	1										
(5) Piscidic acid	-.437	.776**	.956**	-.100	1									
(6) Betanin	.900**	.073	-.258	-.433	-.429	1								
(7) Isobetanin	.929**	.074	-.271	-.422	-.457	.996**	1							
(8) IG1[1]	-.284	.804**	.865**	-.119	.917**	-.245	-.285	1						
(9) IG2[2]	-.347	.685**	.736**	-.021	.826**	-.300	-.344	.969**						
(10) IG4[3]	-.240	.638**	.647**	-.060	.726**	-.114	-.169	.928**	.968**	1				
(11) IG5[4]	.177	.402	.360	-.277	.309	.510*	.449	.465	.401	.583*	1			
(12) Ascorbic acid	.255	.404	.450	-.488	.340	.213	.224	.093	-.140	-.194	.149	1		
(13) Total betalains	.886**	.041	-.299	-.287	-.476	.988**	.986**	-.284	-.326	-.136	.489	.144	1	
(14) Total phenolic compounds	-.433	.779**	.956**	-.102	.999**	-.420	-.449	.921**	.831**	.734**	.322	.336	-.467	1

Statistically significant correlations were determined by Pearson's test (*$p \leq 0.05$, **$p \leq 0.001$) bilateral (n=24). [1]Isorhamnetin glucosyl-rhamnosyl-rhamnoside (IG1). [2]Isorhamnetin glucosyl-rhamnosyl-pentoside (IG2). [3]Isorhamentin glucosyl-pentoside (IG4). [4]Isorhamnetin glucosyl-rhamnoside (IG5).
